# Supplementary material for: Differentiating Benign from Malignant Thyroid Tumors by Kinase Activity Profiling and Dabrafenib BRAF V600E Targeting
Source: Cancers (Basel). 2023 Sep 8;15(18):4477. doi: 10.3390/cancers15184477 (PMC10527361; doi:10.3390/cancers15184477)
Supplement: Supplementary file 1 [file cancers-15-04477-s001.zip › cancers-2436906-supplementary figures.pdf]

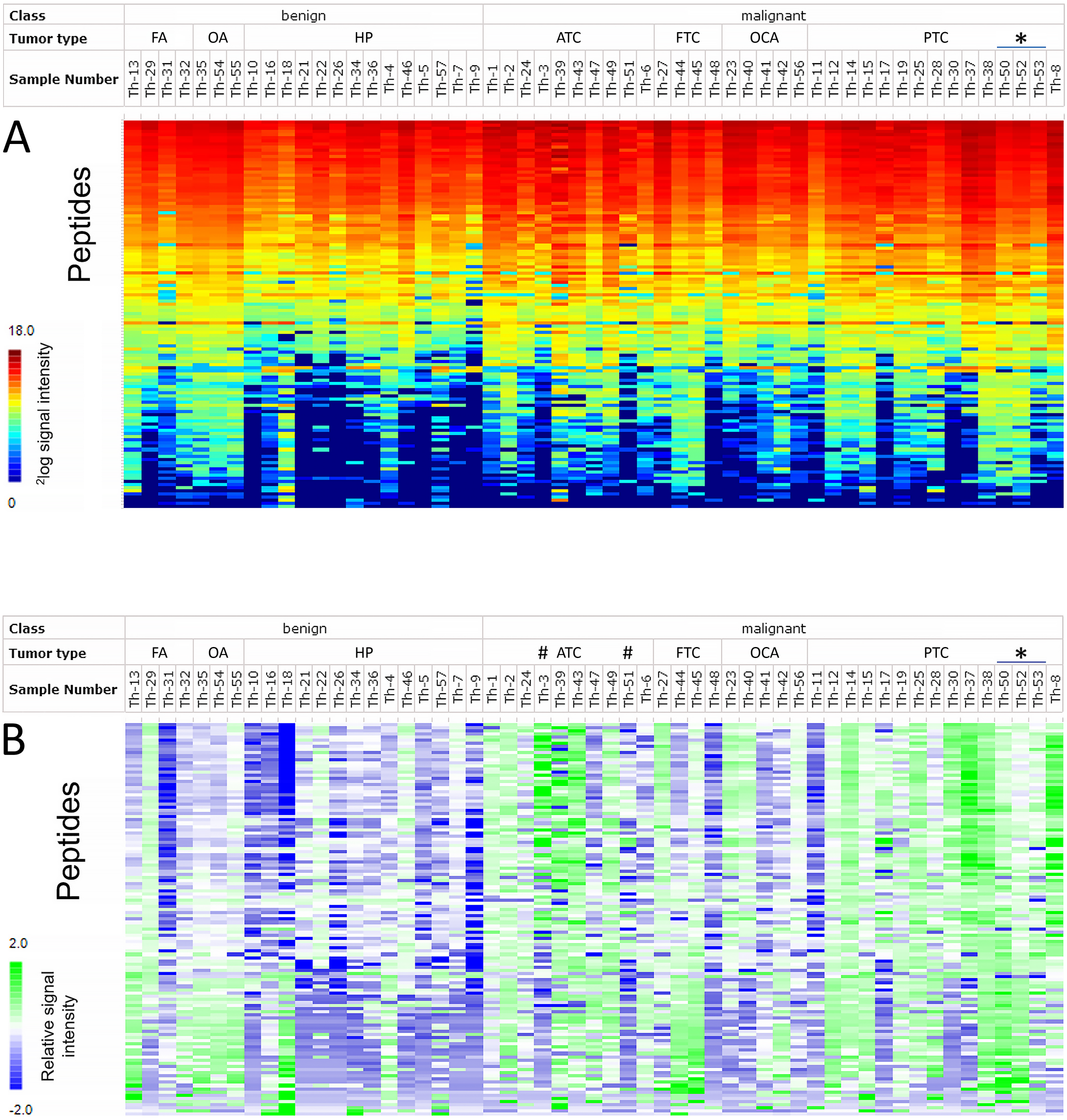

Figure S1. A. Heat map of basal serine/threonine kinase activity profiles for the 55 benign and malignant thyroid tumor samples, arranged by histological subtype. Each column represents a sample, each row represents a peptide. For analysis 123 peptides were included. Signal intensities were determined on PamChip® peptide microarrays, corrected for nonspecific binding (signals present in the absence of ATP) and ^{2}log transformed. Peptides have been sorted by average signal intensity. **B**. Relative serine/threonine kinase activity profiles for the 55 thyroid samples. Signal intensities were expressed with respect to the average signal intensity per peptide, range (^{2}log scale) -2.0 to 2.0, i.e. fourfold change in either direction. Green indicates a signal higher than the average for that peptide of all samples, blue lower than the average. Peptide order is the same as in **Supplementary Figure S1A**.

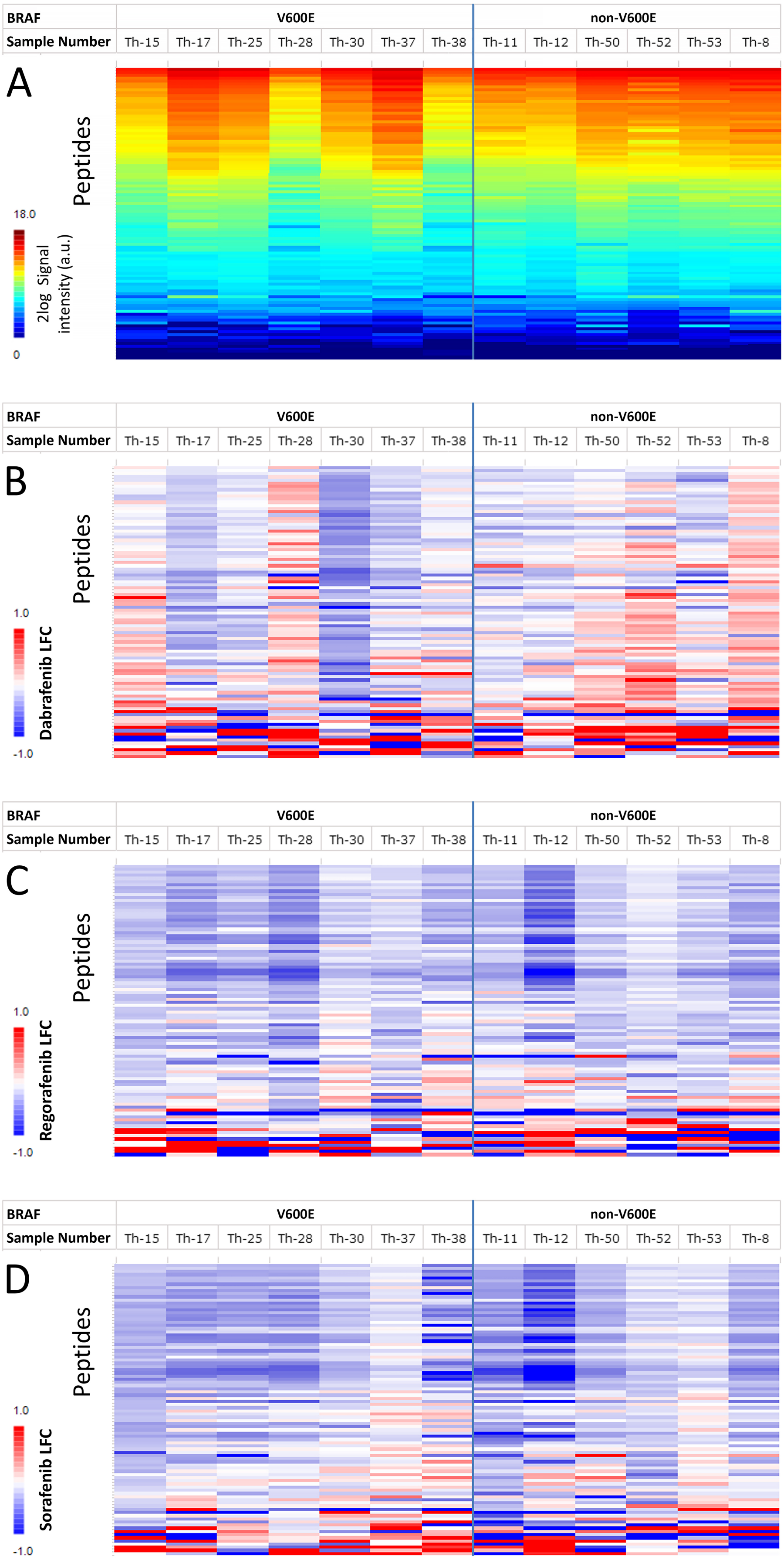

Figure S2. Kinase activity profiles of 13 PTC, i.e. seven *BRAF* V600E-positive and six non-*BRAF* V600E (indicated by non-V600E) thyroid tumor lysates. **A**. Kinase activity profiles without inhibitor (basal profiles). Peptides are sorted by signal intensity, with highest signal on top. **B-D**. Log Fold Change (LFC) of kinase activity profiles of the 13 PTC thyroid tumor lysates after *ex vivo* kinase

inhibition. Inhibition was determined after addition of 10 μM dabrafenib (**B**), 50 μM regorafenib (**C**) or 10 μM sorafenib (**D**) in the assay and signals were compared to the corresponding untreated sample. Peptides are presented in the same order as shown by the heatmap of **Supplementary Figure S2A**. Blue indicates a lower signal than the untreated sample (inhibition), red indicates a higher signal than the untreated sample (activation). Th-50, Th-52 and Th-53 are of the FVPTC subtype.
