# Supplementary material for: Differentiating Benign from Malignant Thyroid Tumors by Kinase Activity Profiling and Dabrafenib BRAF V600E Targeting
Source: Cancers (Basel). 2023 Sep 8;15(18):4477. doi: 10.3390/cancers15184477 (PMC10527361; doi:10.3390/cancers15184477)
Supplement: Supplementary file 1 [file cancers-15-04477-s001.zip › cancers-2436906-supplementary tables.pdf]

Supplementary Table S1. Information of all patients included in the study.

Sample Number	Diagnosis	Oncocytic	Class	Gender	AOD	Recurrent (R) / Non-recurrent (NR)	primary/meta	% tumor cells	necrosis	Molecular genetic typing	w-CIN	gain/loss imbalance	Prediction score	Classification	Inhibitor testing
**Th-13**	FA		benign	F	71	NA	primary	80		NFNV			0.040		
**Th-29**	FA		benign	M	46	NA	primary	50		NFNV			-0.442	FALSE	
**Th-31**	FA		benign	M	57	NA	primary	70		NFNV			0.993		
**Th-32**	FA		benign	F	6	NA	primary	60		NFNV			0.167		
**Th-35**	OA	O	benign	F	37	NA	primary	60		NRAS		loss chr 22	-0.103	FALSE	
**Th-54**	OA	O	benign	F	64	NA	primary	50		NFNV	*	R	0.010		
**Th-55**	OA	O	benign	F	61	NA	primary	50		NFNV	*	R	-0.272	FALSE	
**Th-10**	HP		benign	F	72	NA	primary			ND			0.886		
**Th-16**	HP		benign	M	61	NA	primary			ND			0.708		
**Th-18**	HP		benign	M	51	NA	primary			ND			1.212		
**Th-21**	HP		benign	F	56	NA	primary			NFNV			0.348		
**Th-26**	HP		benign	F	63	NA	primary			ND			0.803		
**Th-34**	HP		benign	F	55	NA	primary			NFNV			0.431		
**Th-36**	HP		benign	F	62	NA	primary			ND			0.372		
**Th-4**	HP		benign	F	62	NA	primary			NFNV			0.143		
**Th-5**	HP		benign	M	77	NA	primary			NFNV			0.682		
**Th-57**	HP		benign	M	13	NA	primary			NFNV			0.576		
**Th-9**	HP		benign	F	56	NA	primary			ND			1.326		
**Th-22**	HP (Graves)		benign	F	54	NA	primary			ND			-0.143	FALSE	
**Th-46**	HP (Graves)		benign	F	48	NR	primary			NFNV			-0.121	FALSE	
**Th-7**	HP (Graves)		benign	F	59	NA	primary			ND			0.132		
**Th-1**	ATC		malignant	F	87	R	meta	100		NFNV			-0.441		
**Th-2**	ATC		malignant	M	43	R	primary	70		IDH1			-0.666		
**Th-24**	ATC		malignant	M	73	R	primary	60		HRAS			-0.209		
**Th-39**	ATC		malignant	F	57	R	primary	30	n	NRAS			-0.714		
**Th-43**	ATC		malignant	F	64	R	primary	30	n	BRAF V600E			-1.008		
**Th-47**	ATC		malignant	M	69	R	primary	30	n	HSPA8 (e9) - MET (i3-e4)			0.203	FALSE	

**Th-49**	ATC		malignant	M	66	R	primary	40		NFNV			-0.736		
**Th-6**	ATC		malignant	F	79	R	meta	50	n	BRAF V600E			0.121	FALSE	
**Th-3**	ATC	O	malignant	M	43	R	primary	80		IDH1, AQP4 (e1) - RAF1 (i8-e9), FGFR3 (e17-i17) - LCN2 (e7)	*	loss (NHG)	-0.499		
**Th-51**	ATC	O	malignant	F	59	R	primary	80		NFNV	*	loss (NHG)	0.331	FALSE	
**Th-27**	FTC		malignant	M	79	R	primary	80		NFNV			-0.561		
**Th-44**	FTC		malignant	F	75	R	meta	30	n	NFNV			0.0963	FALSE	
**Th-45**	FTC		malignant	M	74	NR	primary	70		NFNV			-0.543		
**Th-48**	FTC		malignant	F	36	R	primary	60		NRAS			1.005	FALSE	
**Th-50**	FVPTC		malignant	M	16	NR	primary	70		NFNV			-0.840		X
**Th-52**	FVPTC		malignant	M	47	R	primary	80		NRAS, IDH2			-0.420		X
**Th-53**	FVPTC		malignant	M	39	NR	primary	70		NFNV			-0.444		X
**Th-41**	OCA	O	malignant	M	66	R	primary	60		NFNV	*	loss (NHG)	0.519	FALSE	
**Th-42**	OCA	O	malignant	M	59	R	primary	30		NFNV	*	loss (NHG)	0.120	FALSE	
**Th-23**	OCA (+ ATC)	O	malignant	F	69	UK	primary	20		NFNV			-0.464		
**Th-40**	OCA-MI	O	malignant	F	78	NR	primary	50		NFNV	*	imbalance	-0.239		
**Th-56**	OCA-MI	O	malignant	F	78	NR	primary	70		NFNV			-0.162		
**Th-11**	PTC		malignant	M	80	R	meta	60		NFNV			0.883	FALSE	X
**Th-12**	PTC		malignant	F	42	R	meta	60		NFNV			-0.457		X
**Th-14**	PTC		malignant	M	76	R	meta	70		BRAF V600E			-0.889		
**Th-15**	PTC		malignant	F	56	NR	meta	60		BRAF V600E			-0.299		X
**Th-17**	PTC		malignant	M	45	NR	meta	80		BRAF V600E			-0.070		X
**Th-19**	PTC		malignant	F	21	NR	primary	50		NFNV			-0.335		
**Th-25**	PTC		malignant	M	79	NR	meta	50		BRAF V600E			-0.855		X
**Th-28**	PTC		malignant	F	34	R	meta	20		BRAF V600E			-0.084		X
**Th-30**	PTC		malignant	F	24	NR	primary	50		BRAF V600E			-0.517		X
**Th-37**	PTC		malignant	M	67	R	LN meta	50		BRAF V600E			-1.160		X
**Th-38**	PTC		malignant	F	18	NR	primary	50		BRAF V600E			-1.190		X
**Th-8**	PTC		malignant	F	54	R	meta	50		CCDC6 (e1) - RET (e12), TERTc.-124 C>T			-1.136		X

**Th-49**	ATC		malignant	M	66	R	primary	40		NFNV			-0.736		
**Th-6**	ATC		malignant	F	79	R	meta	50	n	BRAF V600E			0.121	FALSE	
**Th-3**	ATC	O	malignant	M	43	R	primary	80		IDH1, AQP4 (e1) - RAF1 (i8-e9), FGFR3 (e17-i17) - LCN2 (e7)	*	loss (NHG)	-0.499		
**Th-51**	ATC	O	malignant	F	59	R	primary	80		NFNV	*	loss (NHG)	0.331	FALSE	
**Th-27**	FTC		malignant	M	79	R	primary	80		NFNV			-0.561		
**Th-44**	FTC		malignant	F	75	R	meta	30	n	NFNV			0.0963	FALSE	
**Th-45**	FTC		malignant	M	74	NR	primary	70		NFNV			-0.543		
**Th-48**	FTC		malignant	F	36	R	primary	60		NRAS			1.005	FALSE	
**Th-50**	FVPTC		malignant	M	16	NR	primary	70		NFNV			-0.840		X
**Th-52**	FVPTC		malignant	M	47	R	primary	80		NRAS, IDH2			-0.420		X
**Th-53**	FVPTC		malignant	M	39	NR	primary	70		NFNV			-0.444		X
**Th-41**	OCA	O	malignant	M	66	R	primary	60		NFNV	*	loss (NHG)	0.519	FALSE	
**Th-42**	OCA	O	malignant	M	59	R	primary	30		NFNV	*	loss (NHG)	0.120	FALSE	
**Th-23**	OCA (+ ATC)	O	malignant	F	69	UK	primary	20		NFNV			-0.464		
**Th-40**	OCA-MI	O	malignant	F	78	NR	primary	50		NFNV	*	imbalance	-0.239		
**Th-56**	OCA-MI	O	malignant	F	78	NR	primary	70		NFNV			-0.162		
**Th-11**	PTC		malignant	M	80	R	meta	60		NFNV			0.883	FALSE	X
**Th-12**	PTC		malignant	F	42	R	meta	60		NFNV			-0.457		X
**Th-14**	PTC		malignant	M	76	R	meta	70		BRAF V600E			-0.889		
**Th-15**	PTC		malignant	F	56	NR	meta	60		BRAF V600E			-0.299		X
**Th-17**	PTC		malignant	M	45	NR	meta	80		BRAF V600E			-0.070		X
**Th-19**	PTC		malignant	F	21	NR	primary	50		NFNV			-0.335		
**Th-25**	PTC		malignant	M	79	NR	meta	50		BRAF V600E			-0.855		X
**Th-28**	PTC		malignant	F	34	R	meta	20		BRAF V600E			-0.084		X
**Th-30**	PTC		malignant	F	24	NR	primary	50		BRAF V600E			-0.517		X
**Th-37**	PTC		malignant	M	67	R	LN meta	50		BRAF V600E			-1.160		X
**Th-38**	PTC		malignant	F	18	NR	primary	50		BRAF V600E			-1.190		X
**Th-8**	PTC		malignant	F	54	R	meta	50		CCDC6 (e1) - RET (e12), TERTc.-124 C>T			-1.136		X

Supplementary Table S2. *p*-Values of the median serine/threonine kinase activity.

p-Values	FA	OA	HP	ATC	FTC	OCA	PTC
FA		0.125	0.274	0.235	0.932	0.092	0.156
OA			0.061	0.698	0.245	0.9	0.976
HP				**0.008**	0.255	**0.021**	**0.001**
ATC					0.308	0.694	0.633
FTC						0.199	0.178
OCA							0.967
PTC							
